# Astrocytes in Neurodegeneration: Inspiration From Genetics

**DOI:** 10.3389/fnins.2022.882316

**Published:** 2022-06-24

**Authors:** Jingxuan Huang, Chunyu Li, Huifang Shang

**Affiliations:** Laboratory of Neurodegenerative Disorders, Department of Neurology, Rare Diseases Center, National Clinical Research Center for Geriatrics, West China Hospital, Sichuan University, Chengdu, China

**Keywords:** astrocyte, neurodegeneration, gene variant, AD, ALS, PD

## Abstract

Despite the discovery of numerous molecules and pathologies, the pathophysiology of various neurodegenerative diseases remains unknown. Genetics participates in the pathogenesis of neurodegeneration. Neural dysfunction, which is thought to be a cell-autonomous mechanism, is insufficient to explain the development of neurodegenerative disease, implying that other cells surrounding or related to neurons, such as glial cells, are involved in the pathogenesis. As the primary component of glial cells, astrocytes play a variety of roles in the maintenance of physiological functions in neurons and other glial cells. The pathophysiology of neurodegeneration is also influenced by reactive astrogliosis in response to central nervous system (CNS) injuries. Furthermore, those risk-gene variants identified in neurodegenerations are involved in astrocyte activation and senescence. In this review, we summarized the relationships between gene variants and astrocytes in four neurodegenerative diseases, including Alzheimer’s disease (AD), amyotrophic lateral sclerosis (ALS), frontotemporal dementia (FTD), and Parkinson’s disease (PD), and provided insights into the implications of astrocytes in the neurodegenerations.

## Introduction

Neurodegenerative diseases of the central nervous system (CNS) are a group of disorders characterized by chronic progressive damage of neurons with high heterogeneity. Because of the complex mechanisms of neurodegeneration, there are currently no effective treatments. Currently, environmental and genetic factors are both thought to be responsible for neural death. Several studies have confirmed that a large number of genes, such as the apolipoprotein E gene (*APOE*) in Alzheimer’s disease (AD), α-synuclein gene (*SNCA*) in Parkinson’s disease (PD), Cu/Zn-superoxide dismutase 1 gene (*SOD1*) in amyotrophic lateral sclerosis (ALS), and microtubule-associated protein tubulin-associated unit (Tau) gene (*MAPT*) in frontotemporal dementia (FTD), are pathogenic or risk to diseases. The discovery of genes in neurodegenerations have advanced the exploration of disease pathogenesis. Some gene–target therapies have been conducted in clinical research such as antisense oligonucleotide (ASO) silencing for *SOD1* mutation, fused in sarcoma gene (*FUS*) mutation in ALS (Miller et al., 2020; Korobeynikov et al., 2022), as well as targeting protein apoE with antibodies to inhibit amyloid accumulation in AD (Liao et al., 2018). The majority of research on pathogenic or risk gene variants involved in neurodegenerative disorders has focused on neural changes and dysfunction. However, neural dysfunctions, such as oxidative stress damage, protein aggregation, and dysregulated RNA metabolism are insufficient to explain the development and heterogeneity of various diseases. Dysregulation of non-neural cells, such as glial cells, provides an opportunity to gain a better understanding of the pathogenic process of neural death, allowing for more effective target therapies in neurodegenerations.

Astrocytes are the most numerous subsets of glial cells, accounting for at least half of the cells in the brain and spinal cord. Astrocytes are highly differentiated cells that are connected by gap junctions (Liddelow et al., 2020). Astrocytes serve as a barrier, a source of nutrients for neurons, and assist in maintaining synaptic function by regulating ion homeostasis and neurotransmitters. Astrocytes dysfunction, such as reactive and senescent astrocytes that are influenced by both acute stress and chronic inflammatory or degenerative changes, causes the neuron to lose support accelerating neural death (Escartin et al., 2021). Therefore, exploring the relationship between astrocytes and neurodegenerations, particularly pathogenic or risk-gene variants in neurodegenerative diseases, provides a new perspective on disease mechanism.

In this review, we generalized the basic role of reactive astrocytes in neurodegeneration and described the relationships between astrocytes and pathogenic or risk genes in four neurodegenerative diseases (AD, ALS, FTD, and PD). We also discussed the potential roles of astrocytes in the diagnosis and treatment of neurodegeneration.

## The Basic Role of Astrocytes in Neurodegeneration

Astrocytes change morphology and functions in response to CNS injury or disease, known as reactive astrogliosis, and these cells are regarded as reactive astrocytes. Astrogliosis is defined as morphological hypertrophy, proliferation, and increased levels of specific protein markers in astrocytes, such as glial fibrillary acidic protein (GFAP), vimentin, and nestin (Strohm and Behrends, 2020). Reactive astrocytes are classified into two types: A1 for expressing neurotoxic and pro-inflammatory cytokines and A2 for producing neurotrophic factors and recovering synapses. This classification, however, does not cover all conditions and functions of reactive astrocytes. Neurotoxic factors and the loss of supportive functions from reactive astrocytes accelerate neural death *via* different mechanisms (Valori et al., 2014).

First, astrocytes and microglia collaborate in the immune response to stress in CNS. Astrocytes produce chemokines and cytokines that recruit leukocytes and boost local immune responses to protect the surrounding neurons (Castellani and Schwartz, 2020). In response to acute or chronic stress, nuclear translocation of NF-κB, which is activated by microgliosis, induces the inflammatory factors from reactive astrocytes. Meanwhile, reactive astrocytes produce inflammatory factors, such as tumor necrosis factor-alpha (TNF-α), interleukin-1 beta (IL-1β), and IL-17, which accelerates the activation of NF-κB and causes neurotoxicity (Kam et al., 2020; Linnerbauer et al., 2020; Strohm and Behrends, 2020).

Second, astrocytes aid in the maintenance of blood brain barrier (BBB) homeostasis. Astrocytes maintain a portion of their protective effect on BBB integrity by secreting anti-inflammatory factors such as sonic hedgehog, angiopoietin-1, retinoic acid, and insulin-like growth factor-1 to anti-inflammation, and prostaglandins, nitric oxide, or arachidonic acid to regulate cerebrovascular flow (Wei and Shetty, 2021; Zhao et al., 2021). Furthermore, in response to disease, reactive astrocytes play a role in repairing damaged BBB and promoting angiogenesis (Li et al., 2022). Dysregulated reactive astrocytes, on the other hand, destroy the structure of BBB by producing factors to decrease the permeability of BBB. Furthermore, the physical structure of BBB, which is composed of astrocyte end-feet and endothelial cells, is destroyed in reactive astrocytes (Mills et al., 2022). Glial cells-induced neuroinflammation exacerbates BBB damage and reduces its integrity.

Third, rather than neurons, astrocytes perform a large amount of glutamate reuptake *via* transporters such as excitatory amino acid transporter-1, -2 (EAAT-1 and EAAT-2), and glutamate is then metabolized into glutamine in astrocytes (Bantle et al., 2020). Astrocytes also increase glucose metabolism to assist glutamine release from neurons. Astrocytes absorb glucose from blood vessels and produce lactate (Strohm and Behrends, 2020). Mesencephalic astrocyte-derived neurotrophic factor (MANF) is involved in glucose homeostasis and energy metabolism (Wei and Shetty, 2021). Astrocytes protect neurons from fatty acid toxicity and provide energy to neurons *via* fatty acid metabolism (Bantle et al., 2020). Glutamate reuptake (Zhu et al., 2022), glucose (Calsolaro et al., 2021), and lipid metabolisms (Sierri et al., 2021) are all damaged in the active state of astrocytes, resulting in toxicity of excessive glutamate and lipid in neurons.

Fourth, astrocytes maintain synaptic transmission by regulating transmitters and cell excitability (Correa Bernardo et al., 2022). They recycle synaptic transmitters to prevent excitotoxicity to neurons (Strohm and Behrends, 2020). Astrocytes have long-term effects on synapses by releasing growth factors. However, toxicity to synapses was produced in reactive astrocytes *via* several pathways, including ATP deficiency, ion metabolisms dysfunction, and damaged ion channels, all of which degrade the synaptic integrity (Cervetto et al., 2021; Huiliang et al., 2021; Perez-Nievas et al., 2021).

## Astrocytes and Alzheimer’s Disease

Alzheimer’s disease is the most common neurodegenerative disease, characterized by progressive memory loss in clinical manifestation, with misfolding amyloid-beta (Aβ) protein aggregation and hyperphosphorylated tau protein (pTau). In an AD mouse model, pathological changes of microglia appeared before tau protein aggregation (Leng and Edison, 2021). Given that activated microglia influence astrogliosis, astrocytes may play a role in the progression of AD, even in the early and asymptomatic stages. Astrocytes appear neuroprotective in presymptomatic AD cases by internalizing Aβ, whereas astrocyte induced neurotoxicity subsequently due to excessive Aβ aggregation and other neurotoxic factors (de Majo et al., 2020). Biomarkers and pro-inflammatory factors secreted by reactive astrocytes were discovered in cerebrospinal fluid (CSF) of patients with early stages of AD (Janelidze et al., 2018). Astrocyte transcriptome analysis of AD brain tissue revealed gene changes associated with Aβ and pTau pathology in inflammation, protein regulation, oxidative stress, antioxidant function, lipid metabolism, and ion homeostasis (Viejo et al., 2022). Positron emission tomography (PET) and magnetic resonance imaging (MRI) (Choo et al., 2014; Vilemagne et al., 2022a) revealed dynamic changes in reactive astrocytes and Aβ due to mild cognition impairment. At preclinical stages, astrogliosis was induced by early Aβ deposition and loss of gray matter cells (Choo et al., 2014; Vilemagne et al., 2022a), whereas astrocyte atrophy appeared subsequently with greater Aβ deposition (Hsu et al., 2018). Astroglia tracer also revealed that reactive astrogliosis increased and reached a peak in preclinical stages of AD, followed by a decrease of astrogliosis and then rise again in dementia stages, exerting neurotoxic functions (Kumar et al., 2021). These findings suggest that astrogliosis occurs in the early stages of AD, with the formation and process of Aβ, and becomes neurotoxic to neurons as the disease progresses.

### Apolipoprotein E and Astrocytes

The apolipoprotein E (*APOE*) ε4 allele is the strongest risk gene variant of AD, especially late-onset AD, and it is widely expressed in astrocytes. Post-mortem studies discovered that *APOE* ε4 carriers had a higher Aβ plaque burden (Schmechel et al., 1993). Single-nucleus RNA sequencing in the frontal cortex of AD patients with *APOE* ε4 alleles showed astrocytes with highly expressed *APOE* ε4 and markers of reactive astrocytes (GFAP, HSP1B, IFITM3, TAPBP, CHI3L1, etc.) (Table 1; Griswold et al., 2021).

**TABLE 1:** Changes of astrocytes with markers in neurodegenerative diseases with gene variants.

Disease/Gene	Objective	Region	Methods	Astrogliosis/Markers	References
**AD**					
*APOE4* ε4	Human carrying *APOE4* ε4 alleles	Frontal cortex	Single-nucleus RNA sequencing	A1 type astrocytes (GFAP, HSP1B, IFITM3, TAPBP, CHI3L1↑)	Griswold et al., 2021
	Astrocytes from hiPSC with *APOE4*ε4 alleles	–	Cytokine measurement	Increased inflammatory phenotype (IFN-γ, IL-1β, IL-2, IL-4, IL-6, IL-10, IL-12p70, IL-13, and TNF-α↑)	de Leeuw et al., 2022
*PSEN/APP* mutant	APP/PS1 mice	Cortex proximity to Aβ plaques	Immunofluorescence	GFAP↑, cytoskeletal changes	Huffels et al., 2022
**ALS/FTD**					
*SOD1* mutant	Human carrying *SOD1* mutant	Spinal cord and motor cortex	Immunofluorescence	C3↑, astrogliosis	Guttenplan et al., 2020
	Astrocytes from hiPSC *SOD1* mutant	–	Transcriptome-wide analyses	Pan reactive markers (HSPB1, TIMP1, CD44 and OSMR), A1 markers (SERPING1, FBLN5 and GBP2) and A2 markers (S100A10, EMP1, TM4SF1 and CD109)	Taha et al., 2022
*TDP-43* mutant	Mutant *TDP-43* mice	Forebrain	Immunofluorescence	GFAP↑	Bi et al., 2013
	Primary rat astrocytes silencing TDP-43	–	Immunofluorescence, RT-qPCR	CD44, LCN2, FKBP5, and PAI-1↑	LaRocca et al., 2019
*TDP-43* overexpression	*TDP-43* overexpression mice	Frontal cortex	Immunofluorescence, Western blot	GFAP↑	Zamudio et al., 2020
*C9orf72* expansion	Human with *C9orf72* expansion	Spinal cord and motor cortex	Immunofluorescence	C3↑, astrogliosis	Guttenplan et al., 2020
	Poly (GR)_100_ mice	Cortex	Immunofluorescence, RT-qPCR	GFAP↑	Zhang Y. J. et al., 2018
*FUS* mutant	Marmoset silencing *FUS*	Cortex	Immunofluorescence	GFAP↑	Endo et al., 2018
*MAPT* mutant	Human carrying *MAPT* mutant	Frontal cortex	Immunohistochemistry	Astrogliosis with AT8 protein	Erro et al., 2019
**PD**					
*LRRK2* mutant	Human carrying *LRRK2* p.R1441H mutant	Substantia nigra	Immunohistochemistry	Astrogliosis and GFAP↑	Takanashi et al., 2018
	Astrocytes from hiPSC with *LRRK2^G2019S^* mutant	–	Immunofluorescence	GFAP↓, astrocytic atrophy	Ramos-Gonzalez et al., 2021
	Primary mice with *LRRK2^G2019S^* mutant	Substantia nigra	Immunofluorescence	Astrocytic atrophy	Khan et al., 2021
*SNCA*	Monkey with *SNCA^A53T^* mutant	Substantia nigra and frontal cortex	Immunohistochemistry	Reactive astrocytes	Yang et al., 2015
*PRKN* mutant	Human brains and midbrain organoids with *PRKN* mutations	Substantia nigra and frontal cortex	Immunohistochemistry	No reactive astrocytes, GFAP (-), and GFAP↓with disease progress	Kano et al., 2020
*PINK1* mutant	*PINK1* KO mice	Corpus callosum and substantia nigra	Western blot	GFAP↓	Choi et al., 2016
*DJ-1* mutant	Primary astrocytes from *DJ-1* KO mice	–	ELISA	TNFα↓	Ashley et al., 2016
	*DJ-1* KO mice	Striatum	Western blot	Less astrogliosis, GFAP↓	Choi et al., 2018a
*GBA* mutant	Astrocytes from hiPSC of patients with Gaucher disease	–	Immunofluorescence	GFAP and S100β↑	Aflaki et al., 2020

*BBB, blood–brain barrier; AD, Alzheimer’s disease; ALS, amyotrophic lateral sclerosis; FTD, frontotemporal dementia; PD, Parkinson’s disease; APOE ε4, apolipoprotein E ε4 allele; PSEN, presenilin genes; APP, amyloid-beta precursor protein gene; SOD1, Cu/Zn-superoxide dismutase 1 gene; TDP-43, TAR DNA-binding protein 43 gene; C9orf72, chromosome 9 open reading frame 72; FUS, fused in sarcoma gene; MAPT, microtubule-associated protein tau gene; LRRK2, Leucine-rich repeat kinase 2 gene; SNCA, α-synuclein gene; PRKN, parkin gene; PINK1, PTEN-induced putative kinase 1 gene; GBA, glucocerebrosidase gene.*

**FIGURE 1:**
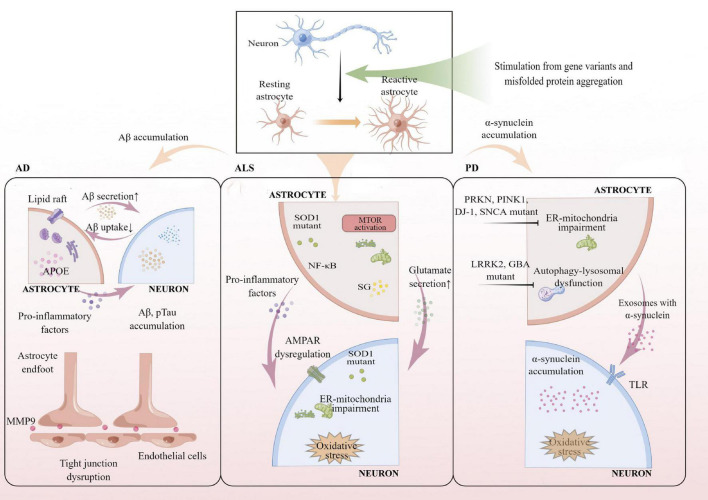
Summary of reactive astrocytes involved in pathogenic or risk gene variants of AD, ALS, and PD. Reactive astrocytes carrying *APOE* ε4 promote Aβ and pTau accumulation in both astrocytes and neurons. Reactive astrocytes with *APOE* ε4 allele secrete pro-inflammatory factors and damage BBB integrity by upregulating MMP9. Reactive astrocytes with *SOD1* mutant increase glutamate secretion and hyperexcitability by dysregulated AMPAR, resulting in ER-mitochondrial impairment and oxidative stress in MNs. α-synuclein accumulation and oxidative stress increase in neurons through damaged ER-mitochondria in reactive astrocytes with *PRKN*, *PINK1*, *DJ-1*, *SNCA* mutant, and autophagy-lysosomal dysfunctions were found in reactive astrocytes with *LRRK2*, *GBA* mutant. AD, Alzheimer’s disease; ALS, amyotrophic lateral sclerosis; PD, Parkinson’s disease; *APOE*, apolipoprotein E; *SOD1*, Cu/Zn-superoxide dismutase 1 gene; *LRRK2*, Leucine-rich repeat kinase 2 gene; *SNCA*, α-synuclein gene; *PRKN*, parkin gene; *PINK1*, PTEN-induced putative kinase 1 gene; *GBA*, glucocerebrosidase gene; MMP9, matrix metalloproteinase 9; NF-κB, nuclear factor-kappa B; mTOR, mammalian target of rapamycin pathway; SG, stress granules; AMPAR, α-amino-3-hydroxy-5-methyl-4-isoxazole-propionic acid receptor; ER, endoplasmic reticulum; TLR, Toll-like receptor.

Astrocytes carrying *APOE* ε4 promote Aβ accumulation both in astrocytes and neurons. *In vitro*, astrocytes from hiPSC carrying *APOE* ε4 allele increased Aβ precursor protein (APP) levels, Aβ secretion in neurons, and decreased Aβ uptake in astrocytes (Lin et al., 2018; Lee S. I. et al., 2021; de Leeuw et al., 2022; Figure 1). Aβ was produced from APP, which was increased by the formation of lipid rafts induced by *APOE* ε4 astrocytes (Lee S. I. et al., 2021). Cholesterol signaling also participates in Aβ accumulation regulation (Wang H. et al., 2021). Astrocytes with *APOE* ε4 increased astrocyte-derived cholesterol (particularly lysosomal cholesterol), resulting in Aβ accumulation (Lin et al., 2018). In addition, astrocytes differentiated from hiPSC with *APOE* ε4 allele showed activation and released inflammatory cytokines, aggravating the pathological process and neuron death of AD (Table 1 and Figure 1; de Leeuw et al., 2022).

In the AD mouse model, removing *APOE* ε4 reduced microglial activation and alleviated Aβ deposition in the cortex (Mahan et al., 2022). Interestingly, knocking out *APOE* ε4 in microglia did not affect Aβ plaque or transcriptional expression compared to controls (Henningfield et al., 2022), indicating that other glial cells, such as astrocytes, play essential roles in Aβ production and accumulation, while microglia appears to maintain Aβ homeostasis. Moreover, *APOE* ε4 in astrocytes participated in pTau aggregation to accelerate AD progression (Wang C. et al., 2021). BBB destruction also hastens the progression of AD. In a mouse model with knockin human *APOE* ε4, the number of astrocyte end-feet covering blood vessels in the cortex decreased, and tight junctions of BBB were damaged by increased matrix metalloproteinase 9 (MMP9) (Jackson et al., 2021; Figure 1).

**FIGURE 2:**
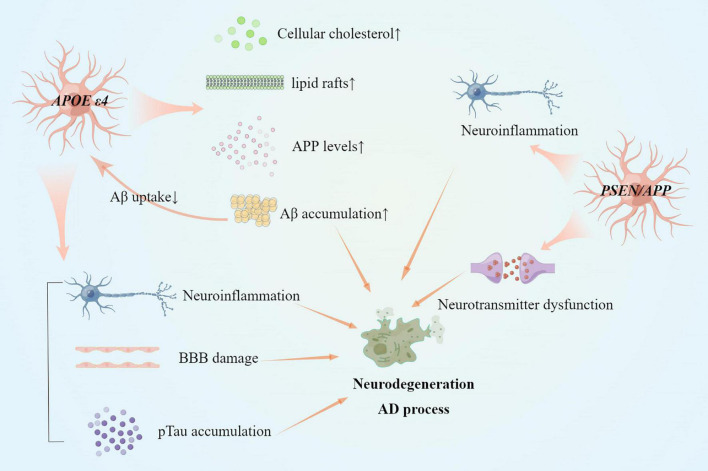
Reactive astrocytes in gene variants of AD and AD pathogenesis. Several mechanisms have changed in astrocytes with *APOE*ε4 allele or *PSEN*/*APP* variant. Both *APOE* ε4 allele and *PSEN*/*APP* variant induce neuroinflammation by pro-inflammatory factors. Cholesterol dysfunction remains in reactive astrocytes with *APOE* ε4 allele. Besides, reactive astrocytes with *APOE* ε4 allele also accumulate Aβ and pTau protein aggregation, and BBB damage.

In conclusion, astrocytes carrying *APOE* ε4 lose their normal functions, resulting in compromised BBB integrity and difficulty in Aβ or pTau clearance, as well as neuroinflammation (Figure 2). With AD progression, reactive astrogliosis occurs in the early stages of AD and precedes the hallmarks of AD (Aβ and pTau deposition).

### Presenilin Genes/Amyloid-Beta Precursor Protein Gene and Astrocytes

Presenilin genes (*PSEN1* and *PSEN2*) and *APP* are responsible for the early onset autosomal dominant inheritance of AD. In the early stages of APP/PS1 transgenic mouse models, astrocytes activate and resist oxidative stress in cellular and extracellular circumstances (Table 1). Astrocytes surrounding Aβ plaque deposits were regulated in K^+^ concentration imbalance to maintain normal functions in neurons and synapses in the early stage (Huffels et al., 2022). *PSEN*/*APP* mutant in astrocytes disrupted amino acid transmission between astrocytes and neurons in response to a long-term detrimental stimulus. Active branched-chain amino acids (BCAA) metabolism, impaired leucine metabolism, and neurotransmitter dysfunction through gamma aminobutyric acid (GABA) uptake capacity were discovered in astrocytes carrying *PSEN1* or *APP* pathogenic variants (Salcedo et al., 2021a,b). In the late stage of APP/PS1 mouse models, astrocytes also triggered immune signaling and lack of neuroprotection (Orre et al., 2014). Reactive astrocytes induced neuroinflammation and activated the transcriptional activity of NF-κB to induce inflammatory factors (Cao et al., 2021; Figure 2).

## Astrocytes and Amyotrophic Lateral Sclerosis/Frontotemporal Dementia

Amyotrophic lateral sclerosis is another neurodegeneration of great concern because of its rapid progression and high mortality. A variety of mechanisms are involved in the pathogenesis of ALS, such as mitochondrial damage, oxidative stress, amino acid toxicity, neuroinflammation, axon transport disorders, endoplasmic reticulum (ER) stress, abnormal protein clearance, abnormal RNA metabolism, etc. The pathological hallmark of the disease is abnormal TDP-43 protein inclusion in the cytoplasm. Familial ALS (fALS) accounts for approximately 5–10% of all cases. The discovery of ALS causative genes assists us to uncover the pathogenesis of ALS. FTD shares some common clinical features and genetics (such as *C9orf72* and *MAPT* mutations). Pathological examination revealed that cortex astrogliosis is a feature of sporadic ALS (sALS) and fALS (Table 1; Kushner et al., 1991; Guttenplan et al., 2020). Astrogliosis detected by PET by measuring monoamine oxidase-B (MAO-B) activity remained in the motor cortex and temporal lobes of patients with ALS and associated with regions lacking cerebral blood flow (Kushner et al., 1991; Higashihara et al., 2021). In several models, astrocyte reaction appeared at the early stages of ALS and FTD and caused motor neurons (MNs) death (Sun et al., 2015; Vahsen et al., 2021). However, the dynamic changes in the reactive astrocytes in patients with ALS remain unclear due to a lack of *in vivo* research on neuroimaging and biomarkers. The mechanisms of ALS causative gene variants in astrocytes may provide insights into the progression of ALS or FTD.

### Cu/Zn-Superoxide Dismutase 1 and Astrocytes

The first causative gene for ALS, Cu/Zn-superoxide dismutase 1 (*SOD1*) mutation, was discovered in 1993. Protein aggregation and prion-like propagation of misfolded SOD1 protein are the main pathologies caused by *SOD1* mutation (Canosa et al., 2022). SOD1 protein is a mitochondrial antioxidant enzyme, and *SOD1* mutation increases cytoplasmic stress granules (SG) and ER stress in neurons (Rajpurohit et al., 2020; Figure 1). *SOD1* gene loss of function contributes to the process of neuron death (Berdyński et al., 2022). A post-mortem study of *SOD1*-ALS patients discovered astrogliosis with a high level of C3 as a marker, as well as astrocyte hypertrophy in the motor cortex and spinal cord (Guttenplan et al., 2020).

Astrocytes derived from human mutant *SOD1*-overexpressing mice specifically damaged MNs, while other types of cells such as interneurons, GABAergic neurons, or dorsal root ganglion neurons were unaffected (Nagai et al., 2007; Bunton-Stasyshyn et al., 2015; Harlan et al., 2019). Meanwhile, in co-culture, microglia and fibroblasts with *SOD1* mutant did not affect the MNs viability (Nagai et al., 2007). Astrocytes with *SOD1* mutation reduced MNs viability in co-culture by inducing nitroxidative stress, transporting oxidative stress *via* secretomes (Rajpurohit et al., 2020), and producing hyperexcitability through dysregulated AMPA receptors and extracellular glutamate secretion to MNs (Van Damme et al., 2007; Rojas et al., 2014; Mohamed et al., 2019; Figure 1). Astrocytes derived from hiPSCs with *SOD1* mutant showed astrocytes activation, increased levels of cytokines and the pro-inflammatory transcription factor NF-κB, and elevated SG. Moreover, astrocytes derived from *SOD1*-mutant hiPSCs stimulated mechanistic target of rapamycin (mTOR) activation induced by increased insulin-like growth factor 1 receptor (IGF1R) levels. IGF1R inhibition in astrocytes was found to be neuroprotective (Granatiero et al., 2021).

Animal models for studying astrocytes with *SOD1* mutation investigated astrocytes changes at different stages. In the early and prodromal stages of ALS, astrocytes in the motor cortex and spinal cord of *SOD1^G93A^* mice demonstrated different vulnerability to oxidative stress as astrocytes in the motor cortex of the *SOD1^G93A^* mouse model had increased the oxidative stress, decreased the antioxidant capacity, and a relative mitochondria respiratory uncoupling, whereas the astrocytes in the spinal cord showed a higher endurance against oxidative damage through an increased antioxidant defense and a preserved mitochondrial respiratory function (Marini et al., 2021). The different responses of astrocytes from the motor cortex and spinal cords to oxidative stress indicate selective damage in ALS progression. Inflammation associated with astrocytes carrying *SOD1* mutant also participates in the prodromal stage of ALS. The increasing level of astrocytic-specific TGFβ1 accelerated the disease progression in the *SOD1^G93A^* mouse model (Endo et al., 2015). The knockout inflammatory factors (IL-1α, TNFα, and C1q) slowed the progression along with reducing toxicity and activation of astrocytes in early mouse models (Guttenplan et al., 2020). Interestingly, decreasing NF-κB in astrocytes from the cortex of *SOD1^G93A^* mice in the prodromal stage inhibited cortical inflammation (Gomes et al., 2019). Despite the fact that astrocytes in the motor cortex and spinal cord had different endurance in the early stage of the disease, widespread and excessive oxidative stress in astrocytes was activated in the late stage of the *SOD1^G93A^* mouse model (López-Blanch et al., 2021). In the *SOD1^G93A^* mouse models, astrocytes from both the motor cortex and the spinal cords were characterized by ER-mitochondrial impairments, which were more pronounced in the mutated motor cortex than in the spinal cord cells (Marini et al., 2021). NF-κB increased in the symptomatic stage as well (Gomes et al., 2019), and astrocyte NF-κB-dependent activation accelerated the disease progression (Ouali Alami et al., 2018).

**FIGURE 3:**
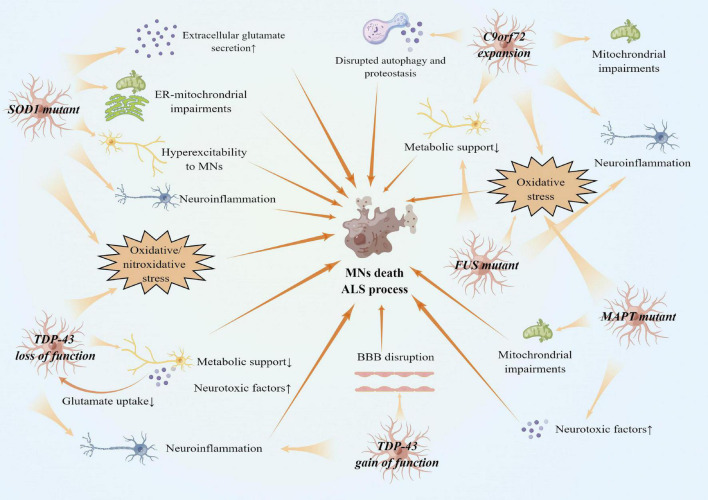
Reactive astrocytes in gene variants of ALS and ALS pathogenesis. There are several faces of astrocytes with gene variants of ALS. *SOD1*, *TDP-43*, *FUS*, *MAPT* variant, and *C9orf72* expansion cause oxidative stress in both astrocytes and MNs. *SOD1* variants also cause astrocytes to secret extracellular glutamates. Reactive astrocytes with *SOD1* variants result in hyperexcitability to MNs. *TDP-43* loss of function in astrocytes reduces metabolic support and glutamate uptake, while *TDP-43* gain of function in astrocytes damages BBB integrity. Other processes, such as mitochondria impairments, neuroinflammation expansion, and disrupted autophagy and proteostasis, also contribute to MNs death.

These findings suggest that *SOD1* mutations participate in reactive astrogliosis in the early stage of ALS. Reactive astrocytes are primarily neurotoxic, causing oxidative stress, excessive excitability, and neuroinflammatory activation (Figure 3).

### TAR DNA-Binding Protein 43 and Astrocytes

TAR DNA-binding protein 43 (*TDP-43*) mutation results in abnormal TDP-43 inclusion in the cytoplasm of MNs and glial cells, which is a pathological hallmark of most sALS and fALS, as well as in FTD (Keating et al., 2022). TDP-43, as a DNA-/RNA-modulating protein, is involved in RNA splicing, transport, and translation, as well as cellular dysfunction and toxicity (Keating et al., 2022). Normal TDP-43 maintains the protective functions of MNs, loss of function of which causes hippocampal and cortical synaptic deficits, as well as dysfunction of RNA metabolisms (Ni et al., 2021). TDP-43 cytoplasmic aggregation in astrocytes is a key feature in fALS with *TDP-43* mutant, sALS, and FTD. TDP-43 positive cytoplasmic inclusions were predominant in astrocytes of the anterior horn of the spinal cord, and quite rare in neurons in a patient with fALS who carried *TDP-43* mutant (Takeda et al., 2019). RNA-seq transcriptomes on post-mortem frontal, temporal cortex, and cerebellum tissue from FTD patients with TDP-43 cytoplasmic aggregation discovered upregulated markers of astrocytes (GFAP) in the frontal cortex (Hasan et al., 2021).

TAR DNA-binding protein 43 mutation, like *SOD1* mutation, is toxic to astrocytes. Astrocytes with TDP-43 mutant showed cytoplasmic mislocalization of TDP-43 protein and decreased astrocyte survival (Serio et al., 2013). When primary astrocytes with *TDP-43* mutation were co-cultured with MNs, MNs death existed as a result of oxidative stress and dysregulated sodium channels (Rojas et al., 2014). On the other hand, normal astrocytes provide neuroprotective and metabolic support to neurons even when TDP-43 aggregation is mislocated. Astrocytes derived from sALS iPSCs were found to reduce cytoplasmic TDP-43 mislocalization from MNs to astrocytes (Smethurst et al., 2020). However, neuronal protection from astrocytes is limited. Primary astrocytes with TDP-43 inclusions accumulated more lipid droplets, activated aerobic glycolysis, and downregulated lactate transporters, resulting in decreased metabolic support for MNs and neurotoxicity (Velebit et al., 2020). TDP-43 inclusion in the cytoplasm also induced astrocyte inflammation and activation, secreting pro-inflammatory factors (IL-1β, IL-6, and TNFα) and causing neurodegeneration and neuroinflammation (Lee et al., 2020; Kim et al., 2021).

A mouse model with a human *TDP-43* mutant that was restricted to astrocytes showed progressive loss of MNs, denervation of skeletal muscles, and consequent paralysis. *TDP-43* mutant activated astrocytes, and glutamate transporters GLT-1 and GLAST in astrocytes were reduced, resulting in MNs neurotoxicity from high glutamate levels (Tong et al., 2013). Besides, astrogliosis and neuroinflammation were found in the majority of the spinal cord and cortex of *TDP-43* transgenic mice (Yang et al., 2022). Levels of mutant *TDP-43* expression in astrocytes determined the degree of injury to MNs (Yamanaka and Komine, 2018). In transgenic mice with *TDP-43* mutant, reactive astrocytes secreted neurotoxic factors, lipocalin 2 (lcn2), to specifically cause neuron death, while other glial cells were unaffected (Table 1; Bi et al., 2013). Oxidative stress and abnormal ATP accumulation in astrocytes were also elevated in *TDP-43* transgenic mice, along with glutathione and L-glutamate uptake deficits (Moujalled et al., 2017; Barton et al., 2020). *TDP-43* overexpression may also activate astrocytes (Table 1). *TDP-43* overexpression in astrocytes triggered inflammation in the brain, resulting in BBB permeability disruption (Zamudio et al., 2020).

These findings suggest that the balanced *TDP-43* expression is essential to maintain astrocyte homeostasis. Astrocytes containing mutant TDP-43 destroy and transform the protective and supportive function of astrocytes into neurotoxicity through secreting pro-inflammatory factors, inducing oxidative stress and excitatory glutamate toxicity (Figure 3).

### Chromosome 9 Open Reading Frame 72 and Astrocytes

Hexanucleotide repeat expansions in *C9orf72* comprise the most common causative gene of ALS and FTD in Caucasians. ER-mitochondrial signaling, protein homeostasis, and RNA metabolisms were found to be dysfunctional in cases with *C9orf72* expansions (Gomez-Suaga et al., 2022). Dipeptide repeat (DPR) polypeptides translated by *C9orf72* repeat expansions and deficiency of *C9orf72* expression are detrimental to neurons and microglia (Lall et al., 2021). Post-mortem analysis of *C9orf72*-ALS patients revealed astrogliosis and astrocyte senescence (Table 1; Guttenplan et al., 2020). A group of enriched proteins specifically expressed from astrocytes was significantly increased when ALS patients with the *C9orf72* expansion were compared to sALS patients (Umoh et al., 2018). GFAP expression was elevated in the motor cortex and hippocampus of transgenic mouse models expressing GFP-poly (GR)_100_ and poly (GA)_50_ (Table 1; Zhang et al., 2016; Zhang Y. J. et al., 2018), whereas GFAP level was not changed in the spinal cord (Schludi et al., 2017). *C9orf72* BAC mouse models revealed that astrogliosis preferred to exist in the end-stage of the disease (Liu et al., 2016). However, there was no difference in plasma GFAP expression between the presymptomatic and symptomatic stages of *C9orf72*-FTD (Heller et al., 2020). Therefore, further research into the specific role of astrocytes with *C9orf72* expansion is required.

Motor neurons undergo cell senescence due to oxidative stress and neurotoxicity when co-cultured with fibroblast-derived astrocytes from *C9orf72*-ALS patients (Birger et al., 2019; Zhao et al., 2020). Both astrocytes and neurons in cortical organoids derived from hiPSCs of *C9orf72* cases existed in DPR polypeptides and expanded RNA foci *via* disrupted autophagy and proteostasis (Mizielinska et al., 2013; Conlon et al., 2016; Zhao et al., 2020; Szebényi et al., 2021). Astrocytes from hiPSCs with *C9orf72* expansion caused voltage-activated currents and action potential output loss in MNs, resulting in disrupted action potentials (Zhao et al., 2020). There was a lack of energy production due to adenosine to inosine deamination defect, reduced glycogen metabolism, and mitochondrial respiration dysfunction in astrocytes in familial *C9orf72* cases (Allen et al., 2019a,b). In addition, intracellular glutamate level was elevated in astrocytes of patients with *C9orf72* expansion (Fomin et al., 2018). In contrast to *SOD1*-ALS astrocytes, glutamate secretion was not increased in *C9orf72*-ALS astrocytes *in vitro* (Mohamed et al., 2019). These results indicated that different glutamate secretion and uptake mechanisms exist in astrocytes with *SOD1* mutant and *C9orf72* expansion.

In conclusion, *C9orf72* expansions are considered the primary cause of toxicity in astrocytes by disrupting energy supply and mitochondrial respiration. Astrocytes dysfunction results in neurotoxicity to MNs and, as a result, ALS pathogenesis (Figure 3).

### Fused in Sarcoma Gene and Astrocytes

Fused in sarcoma gene (*FUS*) mutation mainly exists in early onset ALS and FTD. FUS is a multifunctional DNA/RNA-binding protein involved in RNA processing and metabolism. Cytoplasmic FUS inclusion is another pathological hallmark of ALS. FUS protein mislocalization in mitochondria caused by *FUS* mutation causes mitochondrial death in neurons (Deng et al., 2015). *FUS* mutant also impaired neuromuscular junctions and caused mitochondrial dysfunction in neurons and skeletal muscle (Yu et al., 2022). An ALS patient carrying the *FUS* variant showed increased expression of FUS protein in reactive astrocytes and astrogliosis accumulated surrounding MNs with RNA foci (Wongworawat et al., 2020). Another patient with FTD carrying the *FUS* variant had FUS-positive cytoplasmic or intranuclear inclusion (Murakami et al., 2021). However, FUS inclusion remained mainly in oligodendrocytes rather than astrocytes in other ALS cases with the *FUS* variants (Mackenzie et al., 2011). These disparities were most likely caused by the small number of autopsies performed on ALS/FTD patients carrying *FUS* variants.

Silencing *FUS* expression in the brain by the AAV vector system provoked a proliferation of astrocytes and astrogliosis in marmosets (Table 1; Endo et al., 2018). *FUS* mutant astrocytes triggered MNs susceptible to excitotoxicity *via* AMPAR-mediated cell death (Kia et al., 2018). Clock and clock-controlled genes altered in *FUS*-ALS iPSC-derived astrocytes, contribute to metabolic and redox impairment (Killoy et al., 2021). In *SOD1*-mutant mouse models, misfolding cytoplasmic FUS accumulation in reactive astrocytes caused MN degenerative death *via* pro-inflammatory and neurotoxic pathways such as TNF-α with neutralizing antibodies (Li et al., 2016). These findings indicate that *FUS* deficiency and mislocalization are toxic to astrocytes. Meanwhile, astrocytes with *FUS* mutation reduced neuron viability through neuroinflammation and metabolic dysfunction (Figure 3).

### Microtubule-Associated Protein Tau and Astrocytes

The microtubule-associated protein tau (*MAPT*) is a causative gene in tauopathy diseases such as FTD, and progressive supranuclear palsy (PSP). *MAPT* encodes tau protein and produces six different isoforms of tau, primarily 3R and 4R in approximately 1:1 ratio in adult brains. Post-mortem neuropathological examination revealed that the presence of astrogliosis in patients with *MAPT* P301T variant, with these astrocytes retaining tau protein inclusion (Table 1; Erro et al., 2019).

Tau is expressed by astrocytes and oligodendrocytes, though at lower levels than in neurons (Chung et al., 2021). However, the high expression of phosphorylated tau in astrocytes in the pathological states suggests that astrocytes may also be involved in tauopathy. The overexpression of ptau in astrocytes was sufficient to cause cognitive decline in a mouse model (Richetin et al., 2020). Astrocytes derived from asymptomatic *MAPT* 10 + 16 intronic mutation iPSCs had a higher ratio of 4R:3R-tau transcript and protein than neurons (Setó-Salvia et al., 2021). Reactive astrocytes stimulated by tau express neurotoxic factors and respond to oxidative stress *in vitro* by producing ROS and membrane activation (Esteras et al., 2021; Ungerleider et al., 2021). Tau inhibited mitochondrial calcium efflux in both neurons and astrocytes derived from 10 + 16 *MAPT* hiPSC (Britti et al., 2020). In the presence of mitochondrial dysfunction, astrocytes and neurons derived from 10 + 16 *MAPT* hiPSC were more susceptible to calcium-induced caspase 3 activation and cell death (Britti et al., 2020).

### Astrocytes and Parkinson’s Disease

Parkinson’s disease is the second most common neurodegeneration, characterized by dopaminergic neurons (DA neurons) death in the substantia nigra (SN) and dopamine (DA) deficiency in the striatum. Astrocyte response to acute and chronic stress earlier than neuron in PD. In contrast to the widespread astrogliosis seen in AD and ALS, the cortex of sporadic PD lacked or contained only mild GFAP-positive astrocytes in post-mortem examination (Tong et al., 2015; Cardinale et al., 2021). Patients with PD showed increased reactive astrocytes in the brainstem in the early stage of PD, while decreased reactive astrocytes in the cortex and brainstem in the middle and late stages of the disease, according to an *in vivo* PET imaging study (Wilson et al., 2019). These findings indicate different conditions of astrocytes during the PD process. Astrocytes provide neuroprotection *via* releasing antioxidants in response to oxidative stress in the early stages of PD (Takahashi and Mashima, 2022). However, pro-inflammatory factors, energy deficiency, and synapse dysfunction from reactive astrocytes accelerate neuron death in the late stages of PD (Liddelow et al., 2017; Kam et al., 2020). Pathogenic and risk genes associated with PD may assist in understanding the role of astrocytes in PD.

### Leucine-Rich Repeat Kinase 2 and Astrocytes

Leucine-rich repeat kinase 2 (*LRRK2*) is involved in sporadic and familial PD and is a link to lysosomal and mitochondrial functions. Excessive activation and phosphorylation of LRRK2 protein impaired autophagy in patients with PD (Di Maio et al., 2018; Pang et al., 2022). Astrogliosis and neural loss were discovered in the SNs of patients with *the LRRK2* variant (Table 1; Takanashi et al., 2018).

Astrocyte atrophy was found in the derived astrocytes from PD patients with *LRRK2^G2019S^* mutation (Table 1; Ramos-Gonzalez et al., 2021). *LRRK2^G2019S^* mutated astrocyte from hiPSC appeared to cause nutritional damage to the DAergic neurons, damage the ATP supply, low-mitochondrial density, and ER disorder (de Rus Jacquet et al., 2021). Moreover, autophagy dysfunction was observed in astrocytes derived from *LRRK2^G2019S^* mutated iPSCs (di Domenico et al., 2019; Figure 1). *LRRK2^G2019S^* mutated astrocytes decreased the capacity to internalize and degrade fibrillar α-synuclein *via* the lysosomal pathway (Streubel-Gallasch et al., 2021). These disorders lead to a lack of α-synuclein internalization and clearance in astrocytes, as well as α-synuclein accumulation.

Primary cilia loss and astrocytic atrophy are the main characteristics of astrocytes in *LRRK2* mutant models (Table 1; Khan et al., 2021), with astrocytes losing physical functions, such as ER stress, mitochondrial dysfunctions (Lee J. H. et al., 2021), etc. However, primary astrocytes transformed into reactive astrocytes in *LRRK2* transgenic mice when exposed to additional oxidative stress, such as a toxic dose of MPTP (Arbez et al., 2020). In *LRRK2* mutation mouse models, reactive astrocytes protect neurons through anti-inflammatory functions. Astrocyte activation of Nrf2 inhibited neural degeneration in transgenic mouse models by antagonizing *LRRK2^G2019S^*-induced Mad/Smad signaling (Lin et al., 2021).

**FIGURE 4:**
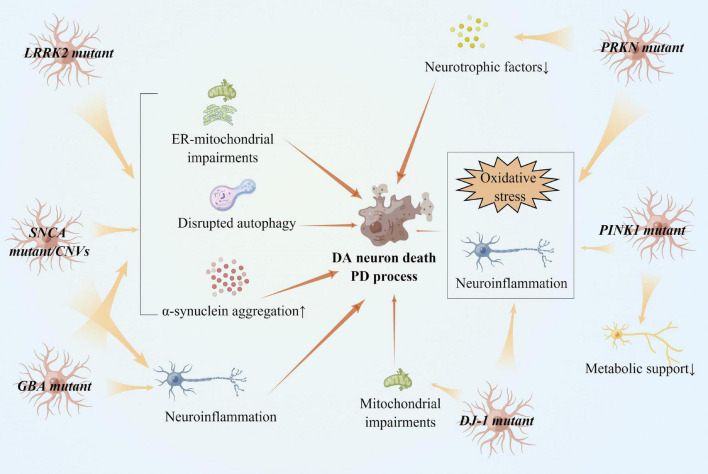
Reactive astrocytes in gene variants of PD and PD pathogenesis. *LRRK2*, *SNCA*, and *GBA* variants impaired ER-mitochondria in astrocytes, accelerating α-synucleins aggregation in dopaminergic neurons. *SNCA* and *GBA* variants also cause neuroinflammation in astrocytes. *PRKN*, *PINK1*, and *DJ-1* loss of functions induces oxidative stress in astrocytes and promote dopaminergic neuron death. Astrocytes lacking *PRKN* and *PINK1* provide less metabolic support to neurons.

These findings suggest that *LRRK2* mutant trigger astrocyte senescence, resulting in neural death, while reactive astrocytes may also play anti-inflammation roles in PD patients with *LRRK2* mutant (Figure 4).

### α-Synuclein Gene and Astrocytes

The α-synuclein gene (*SNCA*) was discovered in 1997 as the first familial PD gene. Point mutations (such as A53T, A30P, E46K, and H50Q) and copy number variation were common pathogenic variants in PD (Polymeropoulos et al., 1997). Insoluble α-synuclein accumulation is an important pathologic marker in PD. Astrocytes in post-mortems were found to internalize a significant amount of α-synuclein fibrils (Kovacs et al., 2014), and to participate in the spread of α-synuclein *via* extracellular vesicles or exosomes (Rostami et al., 2020).

Extensive reactive astrocytes were discovered in *SNCA* A53T transgenic mouse models (Table 1; Yang et al., 2015). In astrocytes, *SNCA* A53T and A30P variants remained in the impaired ER stress. Interestingly, rotenone-induced astrocyte senescence led to axonal degeneration of midbrain neurons with *SNCA* locus duplication (Simmnacher et al., 2020). Cytoplasmic α-synuclein in neurons recruits and activates astrocytes *via* a prion-like process, which is a common pathology in patients with PD (Sznejder-Pachołek et al., 2017; Rizor et al., 2019). Astrocytes reduced α-synuclein proteins misfolding, prevented neurotoxicity from α-synuclein aggregation, rescued damaged DA neurons, and then protected DA neurons at an early stage of PD (Jewett et al., 2018; Zhang Z. et al., 2018). Long-term α-synuclein stress, on the other hand, disrupts the physiological functions of astrocytes. Dysfunction of the ER-Golgi compartment α-synuclein overexpressing astrocytes may lead to a decrease in glial cell-derived neurotrophic factor (GDNF) level, which would suppress the neurite outgrowth (Liu et al., 2018). Besides, exogenous overexpression of α-synuclein proteins in astrocytes damaged the autophagy-lysosomal pathway and promoted astrocyte apoptosis (Erustes et al., 2018). Neuroinflammation induced by astrocytes with α-synuclein aggregation also hastens the progression of PD (Kim et al., 2018; Kwon et al., 2021).

In conclusion, *SNCA* variants cause astrocyte reaction and atrophy, as well as ER dysfunction. Aggregation of α-synuclein is toxic to astrocytes *via* multiple pathways, resulting in astrocyte dysfunction and accelerating α-synuclein accumulation (Figure 4).

### PTEN-Induced Putative Kinase 1/Parkin/*DJ-1* and Astrocytes

PTEN-induced putative kinase 1 (*PINK1*), parkin (*PRKN*), and *DJ-1* genes are all known to play a role in early onset PD (EOPD) (Bonifati et al., 2003; Dawson and Dawson, 2010; Kawajiri et al., 2011). Mitophagy is mediated by these genes *via* the PINK1/parkin pathway. PINK1 protein is a mitochondrial kinase, parkin is an E3 ubiquitin ligase, and DJ-1 protein participate in proteasome degradation (Panicker et al., 2017; Li et al., 2021).

Reactive astrocytes in iPSCs induced from patients with homozygous *PRKN* variants were reduced (Table 1; Kano et al., 2020). Deletion of the *PRKN* leads to abnormal astrocyte function, resulting in DA neurons being vulnerable to oxidative stress (Solano et al., 2008). Parkin controls neuronal homeostasis by regulating astrocyte ER stress and inflammation. In response to ER stress, parkin deficiency astrocytes increased ER stress and released cytokine, and decreased neural support (Singh et al., 2018).

Reactive astrocytes increase PINK1 protein expression (Table 1; Choi et al., 2016; Jarazo et al., 2022). Astrocytes with *PINK1* loss of function increased neuroinflammation and lacked physiological support to neurons (Leites and Morais, 2021). *PINK1* deficiency disrupted mitophagy, altered *PINK1*-dependent ubiquitin phosphorylation, and increased nitric oxide production in astrocytes (Sun et al., 2018; Barodia et al., 2019; Komilova et al., 2021).

*DJ-1* is a neuroprotective protein in PD, which deficiency impaired glutamate uptake into astrocytes by altering EAAT2 expression *in vitro* (Kim et al., 2016). *DJ-1* was found to be highly expressed in reactive astrocytes *in vivo* (Frøyset et al., 2018). *DJ-1* is a positive regulator of STAT3 activation, the most important astrogliosis mediator (Choi et al., 2018b). *DJ-1* overexpression in astrocytes protected neurons from multiple PD processes (de Miranda et al., 2018), and regulated several proteins that support physiological functions on astrocytes and neurons, including redox regulation, anti-inflammation, and mitochondrial respiration (Ashley et al., 2016; Frøyset et al., 2018). *DJ-1* dysfunction resulted in the harmful inflammatory response in PD development (Choi et al., 2019). *DJ-1* knockout astrocytes may provide less neuroprotection to surrounding neurons due to changes in pro-inflammatory mediator expression (Table 1; Ashley et al., 2016). *DJ-1* knockout mice had defective astrogliosis caused by decreased CCL2, Sox9 expression, and reduced monocyte infiltration, which disrupted recovery from CNS injury and accelerated PD progression (Table 1; Choi et al., 2018a,^2020^).

Taken together, since *PRKN*/*PINK1*/*DJ-1* genes are associated with mitophagy and ubiquitin functions, their loss of functions of these genes in astrocytes participates in excessive oxidative stress, neuroinflammation, and mitochondrial dysfunctions, all of which contribute to the PD process (Figure 4).

### β-Glucocerebrosidase Gene and Astrocytes

β-glucocerebrosidase gene (*GBA*), a pathogenic gene for Gaucher’s disease (GD) (Neumann et al., 2009), is the most common genetic risk factor of PD and is associated with an autophagic-lysosomal pathway (Senkevich and Gan-Or, 2020). *GBA* deficiency may cause α-synuclein aggregation and alter neuronal susceptibility to pathology (Henderson et al., 2020; Pang et al., 2022).

Extensively reactive astrocytes with GFAP, S100β, and severe cytoskeletal hypertrophy in astrocytes were discovered from iPSCs from GD patients (Table 1; Aflaki et al., 2020). Furthermore, residual GCase activity appeared to determine the degree of astrogliosis, inflammatory response, Ca^+^ dysfunction, and ability to process α-synuclein (Aflaki et al., 2020; Sonninen et al., 2020). Autophagy and lysosomal storage disorder in reactive astrocytes, combined with inflammation, disrupt mitochondrial homeostasis and cause α-synuclein aggregation in the cortex (Di Malta et al., 2012; Osellame et al., 2013; Booth et al., 2017; Sanyal et al., 2020). These findings suggest that GCase deficiency in *GBA* mutant astrocytes is likely a trigger of reactive astrogliosis through inflammation, impaired autophagy, etc., and as a result of PD progression (Figure 4).

### Further Prospective of Astrocytes in Neurodegeneration

As previously discussed, reactive astrogliosis is common in neurodegenerative diseases with pathogenic or risk gene variants, indicating that astrocytes activate in the early stages and precede hallmarks, and exert different effects throughout the disease progress. New technologies have emerged to assist researchers in directly observing the progression of reactive astrogliosis in patients. The detection of reactive astrocytes is gradually being implemented to detect the disease at its early stages and track its progression. A selective monoamine oxidase-B (MAO-B) tracer is also used to detect reactive astrogliosis since MAO-B is overexpressed in reactive astrocytes. The tracer identified the reactive astrogliosis in mild cognitive impairment (MCI) and AD (Vilemagne et al., 2022b) when compared to controls, and it was detectable at the preclinical stages of Aβ accumulation (Vilemagne et al., 2022a). Markers secreted by reactive astrocytes could also be implemented as a diagnostic tool in patients. Plasma GFAP differs in FTD and AD, which is useful to distinguish FTD and AD and predicting cognitive decline when combined with plasma Nfl detection (Zhu et al., 2021). Salivary GFAP is also considered a potential biomarker for the diagnosis of MCI and AD (Katsipis et al., 2021). These findings indicate that astrocytes are useful to recognize neurodegenerations, though the specificity and sensitivity of reactive astrocytes for diagnosis need to be improved since shared mechanisms induced by astrocytes exist in various neurodegenerative diseases. Moreover, given the neurotoxic roles of reactive astrocytes, regulating pro-inflammatory factors, synapse dysfunctions, high BBB permeability, etc. induced by reactive astrocytes probably reduce the neurotoxicity. Targeting molecular or pathways associated with astrocytes such as STAT, EAAT, GFAP, and connexin 43 *via* adeno-associated virus (AAV) reduced Aβ accumulation in mouse models (Price et al., 2021). Researchers also attempted to restore physiological capabilities in ALS patients by transplanting astrocytes derived from human embryonic stem cells (ClinicalTrials.gov Identifier: NCT03482050).

## Conclusion

Astrocytes are crucial in the pathogenesis of neurodegenerations. Astrocytes maintain and support the physiological functions of neurons, synapses, and BBB. Pathological and related genes of neurodegenerations disrupt astrocyte homeostasis and cause astrocyte activation. Disease models involving several genes (*APOE* ε4 and *SOD1*) revealed that astrocytes activated adaptively to provide neuroprotection in the early stages or even prodromal stages of the disease, whereas reactive astrocytes gradually became neurotoxic as the disease progressed. Different causative genes lead to various pathological processes in astrocytes, including neuroinflammation, oxidative stress, and ER-mitochondria impairment, which eventually lead to misfolding protein aggregation in neurons and neural death. However, due to the heterogeneities of astrocytes in different stages of the disease, astrocytes treatments need to be more cautious. Better treatments based on dysregulated astrocytes require further research into astrocytes targeting neurodegeneration pathologies.

## Author Contributions

JH and CL selected studies and drafted the manuscript. HS made the study design and revised the manuscript. All authors contributed to the article and approved the submitted version.

## Conflict of Interest

The authors declare that the research was conducted in the absence of any commercial or financial relationships that could be construed as a potential conflict of interest.

## Publisher’s Note

All claims expressed in this article are solely those of the authors and do not necessarily represent those of their affiliated organizations, or those of the publisher, the editors and the reviewers. Any product that may be evaluated in this article, or claim that may be made by its manufacturer, is not guaranteed or endorsed by the publisher.
